# Integrative Profiling of Bee Communities from Habitats of Tropical Southern Yunnan (China)

**DOI:** 10.1038/s41598-017-05262-8

**Published:** 2017-07-13

**Authors:** X. W. Liu, D. Chesters, Q. Y. Dai, Z. Q. Niu, P. Beckschäfer, K. Martin, C. D. Zhu

**Affiliations:** 1 Key Laboratory of Zoological Systematics and Evolution, Institute of Zoology, Chinese Academy of Sciences, 100101 Beijing, China; 2 University of Göttingen, Chair of Forest Inventory and Remote Sensing, Büsgenweg 5, 37077 Göttingen, Germany; 3 University of Hohenheim, Institute of Plant Production and Agroecology in the Tropics and Subtropics, Stuttgart, Germany

## Abstract

Understanding and managing pollination service is hindered by taxonomic impediments and paucity of data, particularly in the tropics. Herein we apply integrative species delineation and taxonomy to test impacts of land use on the diversity of bee communities within Xishuangbanna (Yunnan, south China), a highly biodiverse tropical region which has undergone extensive land conversion to rubber plantation. 128 Operational Taxonomic Units (OTU) were inferred by an iterative and integrative approach. Bee activity differed significantly across land use samples, although community composition corresponded more to level of vegetation density, when accounting for spatial structure. Species diversity was high in young rubber plantations, although composition overlapped with other species-rich habitats (natural forest edge and river banks), and older plantations (>8 years) showed very low diversity under all measures. Community structures were similar between the natural forest interior and edge, although analysis indicated contrasting drivers of diversity, with clustering in the interior and overdispersion in the forest edge. Further, phylogenetic diversity and derived indices were underestimated when reference data were omitted from analysis. The description of bee communities herein permits more informed choices in land management with respect to ensuring continuation of essential services by bees.

## Introduction

A component in meeting intensifying demands in food production will be ensuring continuation of pollination services^1^. Globally the majority of crops are pollinated by animal visitors, mostly bees^2, 3^. While honey bees (*Apis* sp.) are familiar and ubiquitous pollinators, they are often just supplemental to the efforts of wild insects^4^. Growing evidence points to substantial losses of pollinators in many regions of the world, with most data from temperate regions^5^. Only a limited number of studies have focused on bee diversity in the tropics^6–9^, although forest degradation is accompanied by severe threats to the diversity of native species. This is particularly serious in Southeast Asia, where a major reason for forest loss is the expansion of large-scale rubber monocultures. In studies on the Malay Peninsula, the abundance of stingless bees (Meliponini) in the understory of primary and secondary forest was significantly higher than in more disturbed forest plots^8^. In Borneo, forest disturbance had little effect on the diversity of stingless bees, although community composition changed in relation to the availability of nesting trees and flowering resources^10^. In a landscape with forests, open land habitats and rubber plantations in southern Yunnan (China), Meng *et al*.^11^ recorded highest bee species richness in the remaining natural forest sites. In contrast, Hoehn *et al*.^12^ found that bee density and diversity was lower in primary forests than in open land and agroforestry systems of a landscape in Sulawesi (Indonesia). Limited knowledge on tropical bee ecology and uncertainty in species boundaries stems from a high number of species, few available taxonomic experts and complexities of field work in tropical forests. This is an impediment to further research, and means in many cases it is only practical to sort specimens to morphospecies or identify taxonomically to genus or sub-genus level. Consequently, conclusions are based on data sets of simplified or incomplete records.

Globally, there are more than 20,000 described species of bees (Apoidea: Anthophila) in seven families^13, 14^. Our understanding of bee diversity has been enhanced in recent years following widespread adoption of molecular analysis^15^, while the accumulation of DNA data serves several key purposes that could assist in gaining an understanding of bee ecology. Taxonomically-labeled sequences serve as a reference framework permitting rapid and standardized DNA-based identification^16^, DNA sequences clustered according to similarity can indicate species boundaries^17^, and are amenable to advanced measures of diversity and community similarity that can be used for testing of ecological hypotheses^18^. Further, integrative taxonomy attempts to consolidate these molecular approaches with morphological information^19^, although a lack of standardized protocols in this field has not been satisfactory addressed^20^. Given the potential of community DNA data to aid tropical bee inventorying and monitoring, and given the paucity of DNA data for this key functional group in the Asian tropics, we undertake extensive collecting and sequencing of bees in tropical south China, thereby demonstrating the utility of DNA data for ecological applications.

**Figure 1:**
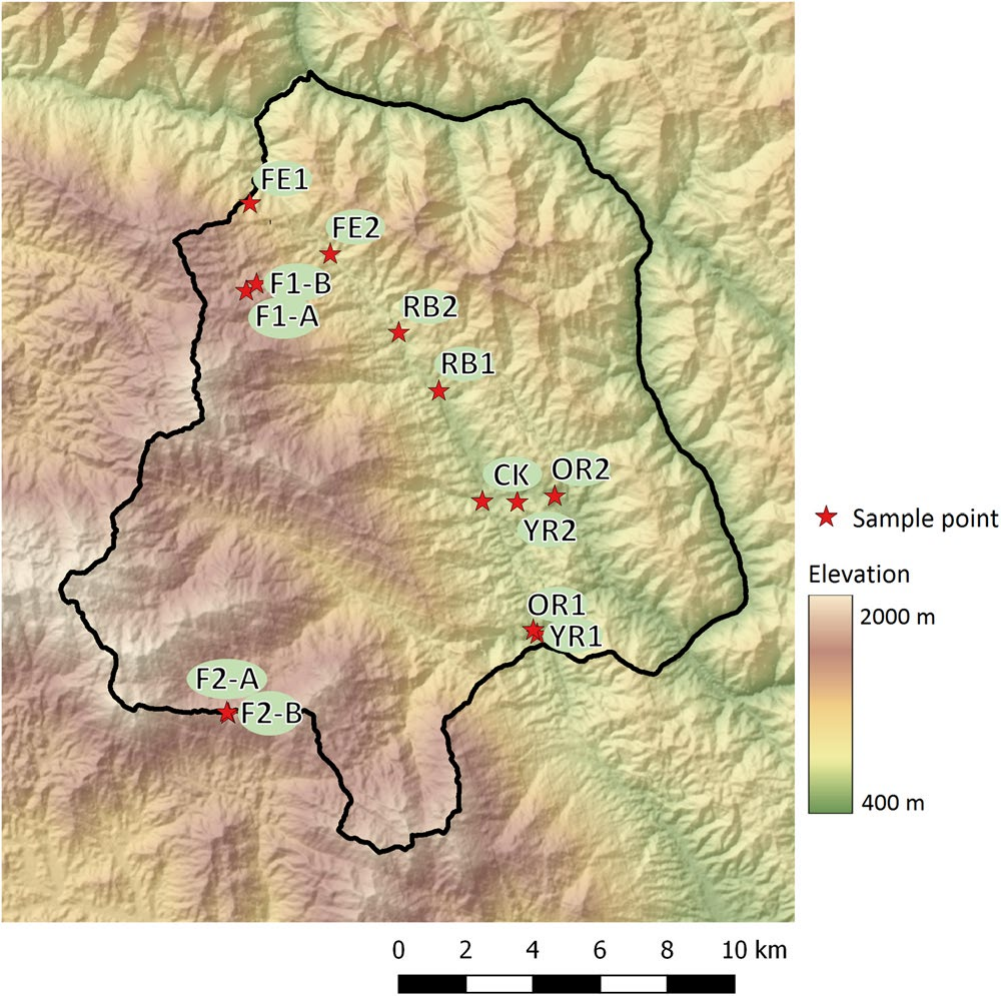
Map of the NRWNNR, showing sample (plot) locations. YR1 = Man-Dian Village (young rubber plantation), YR2 = Man-Fei Village (young rubber plantation), OR1 = Man-Dian Village (old rubber plantation), OR2 = Man-Fei Village (old rubber plantation), FE1 = Guo-Men Mountain (forest edge), FE2 = Ban-Qian-Di (forest edge), RB1 = An-Ma-Xin-Zhai (grassland by river bank), RB2 = Da-Nuo-You (shrubland by river bank), F1-A = Xiao-Nuo-You-Shang-Zhai (forest), F1-B = Xiao-Nuo-You-Shang-Zhai (forest), F2-A = Beng-Gang (forest), F2-B = Beng-Gang (forest), CK = Na-Ban research station. For more information on the location, geography and land use of NRWNNR and Xishuangbanna, see refs 36, 74. Background image: hillshade layer derived from SRTM elevation model. The map was produced using QGIS 2.14.5^75^.

The current research addresses the lack of data on variation in bee communities and of available DNA references for bees in the tropics, by development of a framework of bee species diversity in key habitats of Naban River Watershed National Nature Reserve (NRWNNR) (Fig. 1), a tropical landscape in Xishuangbanna, southern Yunnan, China. We integrate morphological taxonomy, phenetic and phylogenetic analysis of DNA sequences to construct a set of key descriptors of the diversity of tropical bee communities, compare these to traditional approaches (based on morphological identification of specimens) which have previously been used in evaluation of bee diversity and pollination service in Xishuangbanna^11^, and assess how community structure differs through a gradient of land use intensity.

## Results

### Integrative species delineation

**Figure 2:**
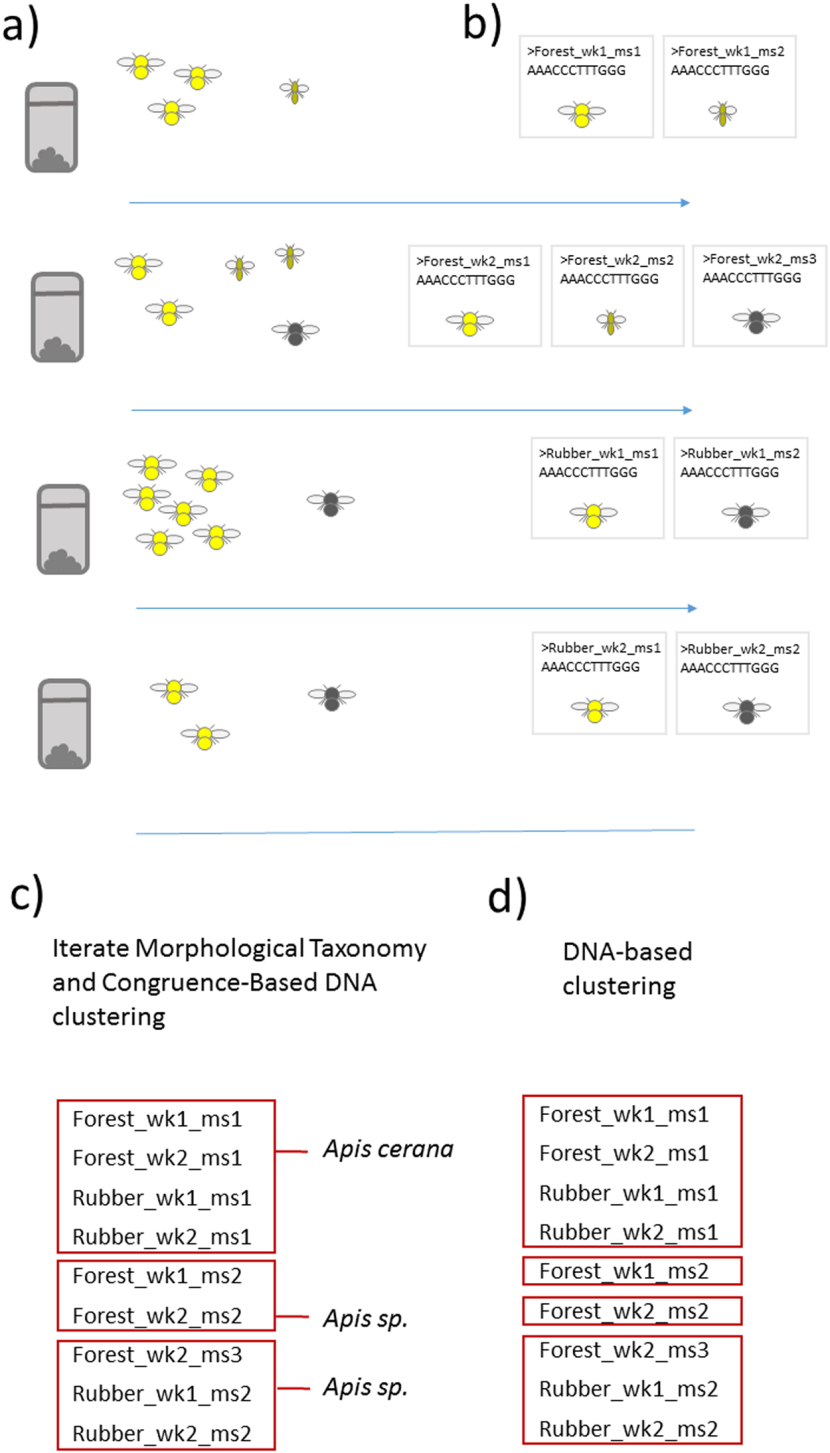
Overview of sorting and taxonomic protocol. (**a**) for each Malaise sample, bees were found and sorted to morphospecies, (**b**) then at least one from each sample/morphospecies is sequenced. (**c**) Morphological taxonomy and clustering of DNA (maximally congruent with species) is iterated (**d**) remaining incongruences are tested according to DNA taxonomy.

Figure 2 depicts the approach used. Collection details are summarized in Table 1 (additional details in Supplementary Table S1). 1837 individual bees (ranging 0–76 and with a mean of 10.1 individuals per Malaise replicate) were found in total, with an average of 4.9 morphospecies per replicate (Fig. 2a). For 748 specimens we successfully sequenced COI, and for 1029 specimens, 28 S rDNA. Successful extraction and sequencing of DNA from bees from Malaise replicates collected on an infrequent (once per > 2 months) basis in the field station (CK) demonstrated little effect of humidity and temperature on DNA quality.

**Table 1:** Sampling details. Column 1 gives sample location (plot) ID, where YR = young rubber, OR = old rubber, FE = forest edge, RB = open river bank, F = natural forest interior.

Plot	Malaise samples	Sampling start date	End date	Total individuals	Average individuals
YR1	16	2014-04-07	2014-08-19	448	28
YR2	15	2014-04-10	2014-08-19	159	10.6
OR1	16	2014-04-07	2014-08-19	44	2.7
OR2	16	2014-04-10	2014-08-19	32	2
FE1	17	2014-04-08	2014-08-18	48	2.8
FE2	17	2014-04-08	2014-08-18	247	14.5
RB1	16	2014-04-09	2014-08-18	235	14.7
RB2	16	2014-04-16	2014-08-18	236	14.7
F1A	12	2014-05-19	2014-08-18	170	14.2
F1B	12	2014-05-19	2014-08-18	65	5.4
F2A	11	2014-05-16	2014-08-17	90	8.2
F2B	10	2014-05-23	2014-08-17	56	5.6

For each study plot, the number of malaise samples is given (Malaise collection replicates; samples usually taking place about once per week), along with the collection start and end dates, number of bee individuals caught in the sample and the average.

Operational Taxonomic Unit (OTU) clustering optimized to morphologically assigned species-boundaries (Fig. 2c) was conducted, a number of errors were corrected during the process, leaving 134–137 OTUs (GTR distance of 0.0055, that with the highest taxonomic congruence; Supplementary Fig. S1). More permissive thresholds (> = 0.006) were noted to lump the abundant and close relatives *Apis cerana* Fabricius and *A. dorsata* Fabricius, whereas more stringent (<0.004) over-split several species. Constraints were applied to enforce clustering of unambiguous taxonomic species. Morphological and DNA-based taxonomic work was conducted during the iterative process. Statistical DNA-based identification of 649 specimens (subsequent to sequence processing and filtering) with COI gave a rate of taxonomic assignment (Pr > 0.95) to family, genus, species, of 95.7% (4 families), 76.4% (13 genera), 22.5% (13 species), respectively. For 777 specimens with 28 S this was 100% (4 families), 73.1% (15 genera), 17.0% (10 species). Morphological taxonomy better targeted diversity, with representatives (238 select specimens) of 92% of all OTU identified at least to genus, finding 32 genera and 36 species among them.

**Figure 3:**
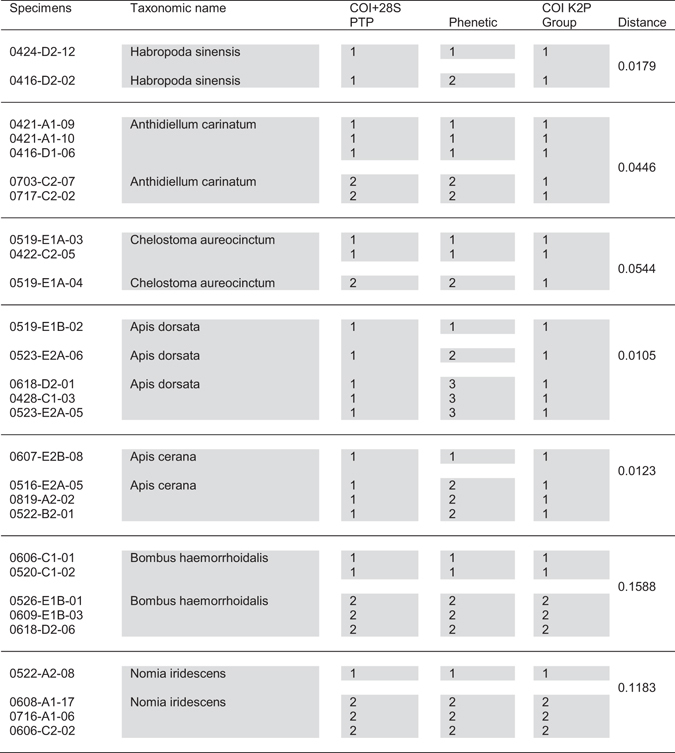
Seven incongruences remained after iterative delineation of OTU. No cases remained of two or more taxonomic species occurring in individual OTU. Molecular evidence was strong that the lower two species, *Bombus haemorrhoidalis* and *Nomia iridescens*, both were composed of two species. Molecular evidence for other cases was not conclusive. Column 1 gives specimen codes; note, where> 3 specimens were present in a OTU, only 3 are shown. Column 2 gives taxonomic name after morphological identification. Columns 3 and 4 give OTU from DNA taxonomy (2 methods) based on the two genes COI and 28 S. Columns 5 and 6 give groupings and K2P distance between specimens under question (split where> 9% divergence^15^). Numbers in columns 3–5 depict different OTU (also by shaded boxes).

Species hypotheses from morphologically-constrained preliminary OTU were tested with several DNA taxonomy methods (Fig. 2d). As for the intrinsic phenetic clustering approach, the point characteristic of a switch to species level groups occurred at a genetic distance of ~0.0033, which suggested 179 OTUs (Supplementary Fig. S1). Clustering was conducted using the phylogeny-based Poisson Tree Process (PTP) method and phenetic clustering of General Time Reversible (GTR) distances. PTP delineated 254 OTUs, although there were topological features that led to substantial overestimation of diversity (Supplementary Fig. S2), specifically, the presence of long branches nested within clusters of otherwise near-identical members led to each of the latter erroneously assigned separate OTU. When accounting for these errors, ~113 OTUs were inferred, with species boundaries mostly congruent with other methods. The seven incongruences remaining after taxonomic iteration were resolved depending on level of evidence from DNA taxonomy (Fig. 3). In two cases (lower two species of Fig. 3), all DNA taxonomy results implied multiple species, and thus OTU were adjusted accordingly, otherwise morphological groupings were retained. 128 Resolved OTUs were used in analysis of community diversity (Supplementary Dataset File, with genus-level summary in Table 2).

**Table 2:** Genus information. For information tabulated for OTUs or individuals, see Supplementary Dataset File.

Genus	OTUs	Young_Rubber	Old_Rubber	Forest_Edge	River_Bank	Forest
**Amegilla**	1	0	0	1	0	0
Anthidiellum	1	2	0	2	1	0
**Anthophora**	1	0	0	0	2	0
Apis	4	59	15	28	49	53
**Bombus**	3	0	0	2	1	25
Braunapis	3	3	0	9	1	0
**Ceratina**	11	21	2	26	11	10
Chelostoma	1	0	0	1	0	2
**Coelioxys**	4	7	1	1	1	0
Ctenoplectra	2	1	0	0	5	3
**Elaphropoda**	1	1	0	2	0	0
Euaspis	1	0	0	1	0	0
**Eucerini**	1	0	0	3	0	0
Habropoda	1	0	0	0	2	0
**Halictus**	1	4	0	8	7	4
Heriades	1	14	0	18	9	0
**Hylaeus**	7	1	0	3	13	7
Lasioglossum	29	58	14	67	65	85
**Lepidotrigona**	1	0	0	1	1	6
Lipotriches	5	13	7	7	23	0
**Megachile**	16	24	2	5	13	3
Meliponini	1	0	0	0	0	1
**Nomada**	1	7	1	1	1	1
Nomia	12	37	1	7	29	0
**Pithitis**	1	14	0	7	10	0
Pseudoanthidium	1	2	0	2	2	0
**Sphecodes**	3	11	0	0	1	1
Tetralonia	1	1	0	0	0	0
**Tetrigona**	1	4	11	3	7	4
Thrinchostoma	1	1	0	0	2	0
**Unidentified (Apoidea spp.)**	10	9	0	6	21	18
Xylocopa	1	0	0	0	1	0

### Community Ecology

**Figure 4:**
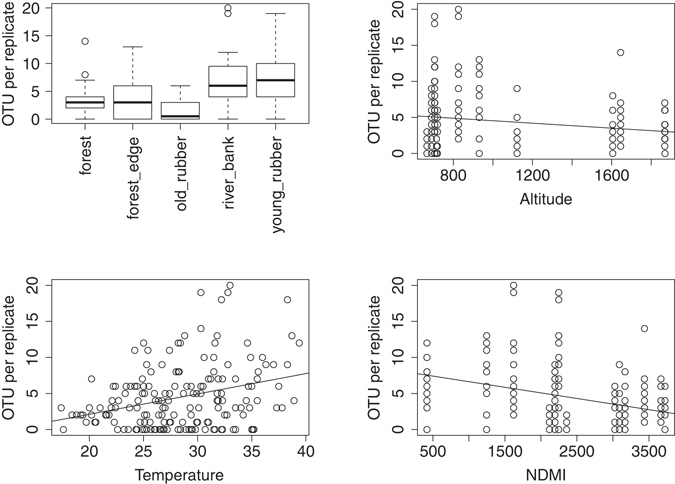
Number of bee MOTU in each malaise replicate, according to (**a**) habitat; (**b**) altitude; (**c**) temperature; (**d**) NDMI (vegetation density index).

**Figure 5:**
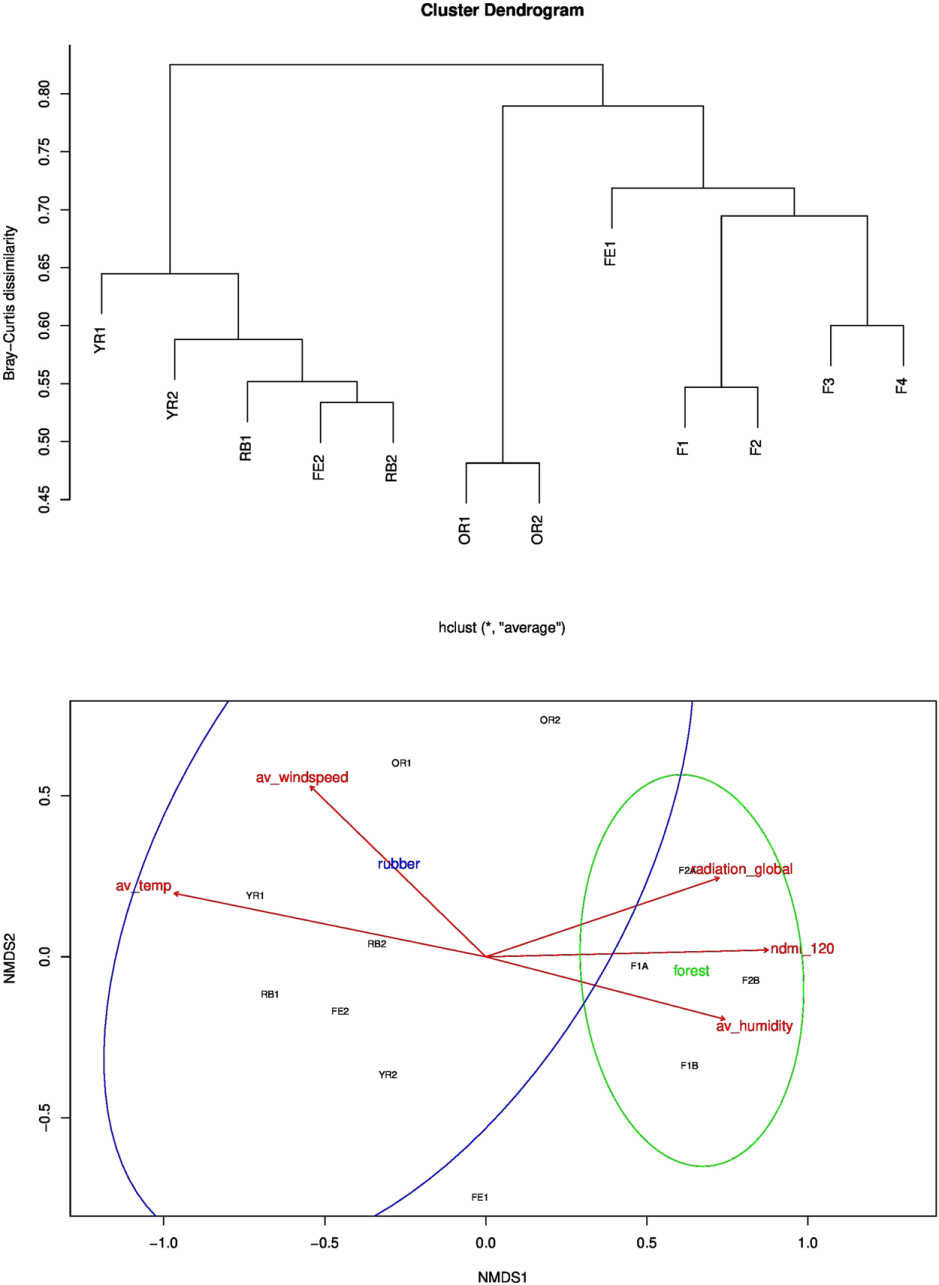
(**a**) Dendrogram depicting difference in bee community structure. YR = Young Rubber. OR = Old Rubber (>8 years). RB = Open River Rank. FE = Forest Edge. F = Forest. (**b**) Multivariate structure visualized with non-metric multidimensional scaling. Area within ellipse is 95% confidence interval for the 4 rubber and 4 forest sites. Also plotted are key environmental variables, particularly average temperature and NDMI (vegetation density index).

**Figure 6:**
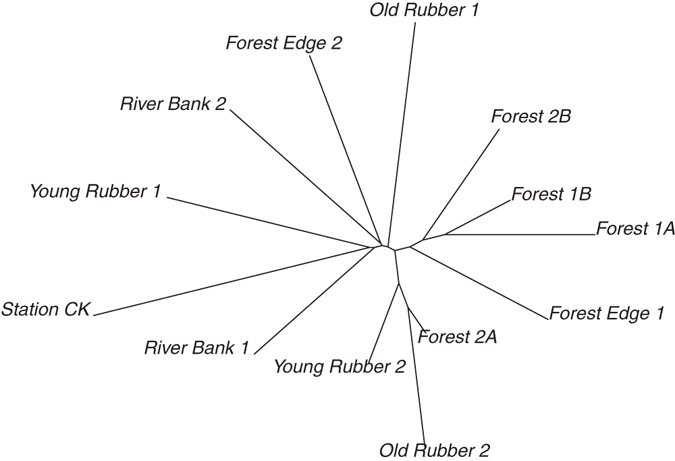
Neighbour joining phenogram depicting differences in phylogenetic community structure of bees between sample locations. The distance between a pair of locations is calculated from phylogenetic distances between their taxa, and weighted by taxon abundances.

For individual (usually weekly) samples, more species were found during the dry season (April to June; p = 0.0013, F value = 10.3, d.f. = 1, Anova), in areas of less dense vegetation (lower NDMI; p =  < 0.001, t value = −4.32), at lower altitudes (p = 0.019, t value = 2.37), with significant differences between habitat types (p = ≪0.0001, F value = 15.8, d.f. = 4, Anova; Fig. 4) and it was indicative that more species were present when temperatures were higher (p = 0.10, t value = 1.6). When pooling over the whole collection effort, species richness, species diversity and phylogenetic diversity was similar at the river bank, young rubber plantation and forest edge (Table 3). Communities were clustered according to Bray-Curtis dissimilarity (Fig. 5a). Composition of communities were more dissimilar between sites of different habitat than sites of the same (Adonis test, 999 permutations, P = 0.01, d.f. = 4; Anosim R = 0.543, P = 0.011, 999 permutations). However, there was a significant spatial component (Mantel r = 0.265, p = 0.03). This was accounted for by removing two (closely located) natural forest samples (Mantel r = 0.223 p = 0.141). Under these conditions, level of vegetation density (NDMI_120; p = 0.028, d.f. = 1, F = 1.97) proved a better correlate of dissimilarity in bee community composition than habitat type (p = 0.061, d.f. = 4, F = 1.54). Besides, composition of communities in the forest were more unique, while plantation communities tended to be those also found in other habitats (Fig. 5b). Beta diversity was also calculated to account for incomplete sampling, and separated into individual richness and replacement components^21^, although neither was dominant (Btotal = 0.822, Brich = 0.407, Brepl = 0.415). Analysis of community phylogenetics within and between habitat types showed significant clustering (Net Relatedness Index or Nearest Taxon Index > 1.96) in sites F1B (forest interior) and RB1 (grassland by river). Conversely, generally lower NRI and NTI are observed at the forest edge. Figure 6 depicts the differences in phylogenetic structure between habitat types, where there are two notable clusters. The first show similarity in community structure of sites YR2 (young rubber), OR2 (old rubber), F2A (forest). The second indicates similarities between sites F1A (forest), F1B (forest) and F2B (forest) and FE1 (forest edge). Further, although less richness was observed in the forest interior, it was structurally most similar in community diversity to the more diverse forest edge.

**Table 3:** Community diversity (various measures of) as compared between sites of differing habitat type.

Plot	nOTU	Div	PD	MPD	NRI	NTI	MNTD
YR1	48	0.956	3.785/6.691	0.3651/0.6213	1.2632/1.4771	0.7667/1.2364	0.1015/0.1486
YR2	27	0.903	2.803/4.928	0.3603/0.4923	1.0795/2.5636	0.3713/1.3356	0.1309/0.1633
OR1	17	0.904	2.075/3.776	0.3631/0.6215	0.7254/0.3619	0.4554/0.5497	0.1505/0.2416
OR2	6	0.679	0.986/2.025	0.3670/0.4432	0.3136/0.4048	0.328/-0.2104	0.2255/0.4527
FE1	18	0.916	2.686/4.502	0.4141/0.5939	−0.8120/1.0304	−0.5344/1.2721	0.175/0.2013
FE2	46	0.951	4.201/7.454	0.3701/0.6183	1.0642/1.4025	-0.3435/0.1821	0.1183/0.1846
RB1	40	0.952	3.293/6.010	0.3528/0.5901	1.8640/2.0921	1.3730/2.2385	0.1007/0.1288
RB2	49	0.962	4.299/7.762	0.3767/0.6368	0.6689/1.3497	0.2912/0.3715	0.1077/0.1761
F1A	22	0.863	2.330/4.210	0.3672/0.5627	0.7536/0.8030	1.1403/0.4895	0.1242/0.2171
F1B	15	0.860	1.302/2.476	0.2865/0.4714	2.8255/2.0305	3.1634/2.0990	0.0769/0.1436
F2A	9	0.767	1.269/2.395	0.3813/0.4424	0.1514/1.1980	0.8234/1.5689	0.1729/0.1967
F2B	15	0.853	1.508/2.682	0.3368/0.5363	1.4289/0.9904	2.2468/1.0855	0.104/0.2105

For each site the values are calculated over all replicates. Abbreviations: nOTU = Number of OTU, equivalent to species richness; Div = Simpsons Diversity Index, calculated from number of individuals of each OTU, and gives probability that two randomly selected individuals will be of the same species; PD = Phylogenetic diversity^76^; MPD = mean phylogenetic distance between a randomly selected pairs; NRI = Net relatedness index (an overall measure); NTI = Nearest taxon index (a measure restricted to the terminals of the tree); MNTD = mean nearest taxon distance, average phylogenetic distance to nearest neighbor (excluding conspecifics), this index has also been referred to as mean nearest neighbor distance. NRI and NTI are standardized forms of MPD and MNTD, respectively, and are significant where >1.96 (clustered) or <−1.96 (dispersed). Two values are given for phylogeny-derived indices, those calculated from tree including new OTU only/calculated from tree including both new OTU and reference data.

**Figure 7:**
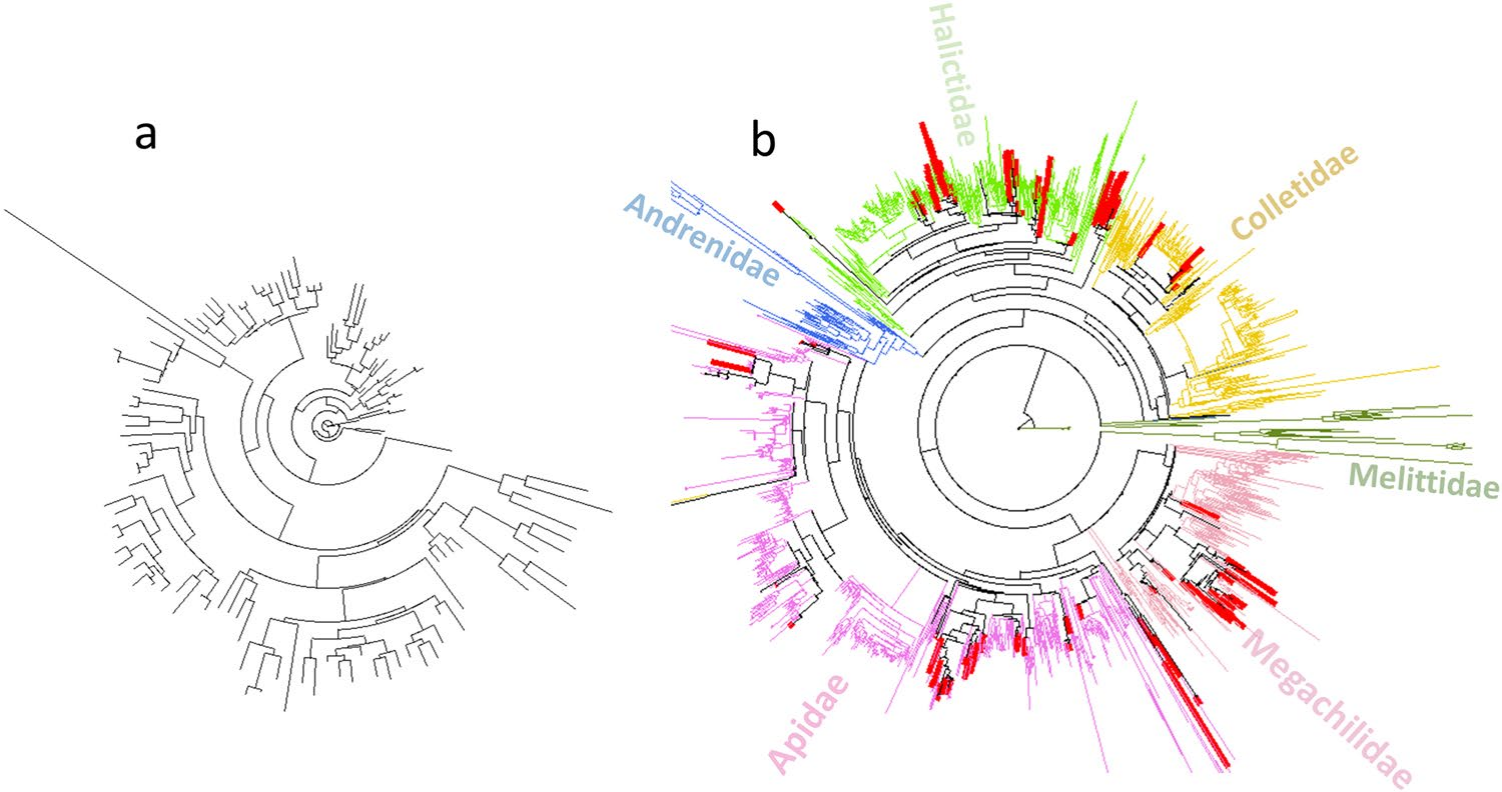
(**a**) Phylogeny only of the 128 OTUs generated in the current study. (**b**) Phylogeny of OTUs as in (**a**), except also including mined reference data, constrained to a published bee tree-of-life. OTUs from bees of NRWNNR sampled herein are indicated in red, six families of the mined reference data are otherwise coloured. Community diversity of habitat subsets of OTUs was calculated in the context of (**a**) and (**b**).

Faith’s phylogenetic diversity (PD), mean phylogenetic diversity (MPD) and mean nearest taxon distance (MNTD) were invariably higher where calculated where species were placed in the context of a more complete phylogeny (Fig. 7b) compared to where calculated from the sampled communities only (Fig. 7a). PD (r = 0.998, p-value < 0.001, Pearson’s product-moment correlation) was correlated where calculated either with or without reference data, as was MNTD (r = 0.814, p = 0.0013). However, the standardized indices NRI (r = 541, p = 0.07) and NTI (r = 0.544, p = 0.067) were not significantly correlated with/without inclusion of reference data.

The patterns in diversity inferred from our 2014 effort were compared to data presented by Meng *et al*.^11^. Species diversity (Simpsons index) calculated for combined counts of our (i) four forest samples, (ii) two young rubber, (iii) two old rubber and (iv) riverbank (grassland and shrubland) sites were 0.902, 0.962, 0.867, 0.967 respectively. Whereas species diversity calculated from data provided by Meng *et al*. for (i) four forest sites, (ii) two young (< = 8 year) rubber, two old (> = 20 year) rubber, and the three open sites (forest clearfell, grassland and shrubland) were 0.929, 0.913, 0.911, 0.890. As for species richness we observed 35, 63, 17, 65 species, compared to 42, 22, 4 and 5 morphologically assigned species reported by Meng *et al*., respective as above.

## Discussion

The iterative/integrative approach proved invaluable in capturing errors (data entry, lab processing, labeling), of which several examples follow. The morphospecies given the preliminary morphological name *Bombus breviceps* Smith was revealed as an assemblage of at least three species after DNA analysis (confirmed during taxonomic iteration). During preliminary molecular analyses we observed clustering of the dwarf honeybee sister species *A. florea* Fabricius and *A. andreniformis* Smith, which were lumped even under stringent clustering settings (see ref. 22). The species are endemic to subtropical Southeast Asia and easy to distinguish morphologically from hair patterns (amongst other characteristics), though notably they have identical 28 S sequences (while distinctive in COI). This lumping of different species was due to a combination of a lack of distinguishing characters in 28 S, and failure to sequence COI in some specimens. Although the rate of species identification for the DNA-based approach was examined, this was largely just for error-checking. All taxonomic labels on the data made available herein were made independently by ourselves (Z.-Q. Niu). Besides, publically available bee reference data are currently lacking for the Asian tropics, DNA barcoding being less informative in these cases.

Following the iterative steps based around morphological identifications, incongruences remained, which were treated as preliminary hypotheses tested through independent delineations made with DNA taxonomy. Genetic differentiation in the ubiquitous honeybee *A. cerana* was manifest as multiple OTUs; high genetic differentiation of *A. cerana* relative to congeners in China has been noted previously^23^. Similarly, a small number of singletons were observed after clustering in the ubiquitous *A. dorsata*, which were manually resolved. These incongruences in delineation were resolved based on weight of evidence. Morphological groupings were retained where evidence from DNA taxonomy to the contrary was not unanimous (particularly *Chelostoma aureocinctum*, *Anthidiellum carinatum* and *Habropoda sinensis*). Two specimens (0520-C1-02 and 0526-E1B-01) were confirmed (morphologically) *Bombus haemorrhoidalis* despite unexpectedly high distance at COI (K2P of 0.84). This species is part of the *Bombus (Megabombus) trifasciatus* species complex, notably highly diverse and in which species boundaries have been disputed^24^. A similar case was observed with two specimens (0608-A1-17 and 0522-A2-08) confirmed as a male and female *Nomia iridescens*, but only 91% identical at COI. Such discrepancies would likely be overlooked under any single evidence method.

Understanding the ecological mechanisms behind composition of communities has recently being facilitated by advanced descriptions of diversity afforded by DNA-based analysis^25^. Use of DNA data permitted testing of community composition using defined criteria for diversity (excepting several adjustments), as opposed to more arbitrary species definitions. Under various measures there was high diversity found at the river bank, young rubber plantation and forest edge (Table 3, columns nOTU and Div). There was often substantial variation between sample pairs, these typically correlated with the levels of disturbance. Of the two rubber plantation samples, that with the greater diversity (site YR1) was notable for a reduced level of management, pesticide use, and less dense plantation. The forest edge site showing the lesser diversity (FE1) was adjacent to a road and frequented by locals. High richness in the plantation compared to forest interior might initially seem unexpected, although it should be noted that a relatively high number of the species found in the young rubber plantation are those also observed in other habitat types, with the most distinctive fauna in the forest edge and river bank. Forests are well established for insect diversity, with bees nesting in dead wood, and farmers local to NRWNNR obtain bee nests for commercial species from the forest (cut wood is placed in the forest and collected some time later). Richness in the forest interior was less that of the forest edge, although the communities could be similar in composition (Figs 5a and 6), indicating forest as the source for insects caught at the edge. We suggest this due to insects in the forest canopy descending to ground level at the margin. Other factors reducing bee capture in the forest interior would be high altitude, low temperature, dense vegetation, fewer flowers at ground level (where trapping occurs). High activity is often reported in open and flower-rich areas^26, 27^, confirmed by analysis of NDMI from RS data herein. Species most abundant and found in all habitats were *Apis cerana* and *Lasioglossum* spp. Under the caveat that results on individual species or genera within a community analysis should be treated with caution (being based on much fewer samples), unique species observed in the forest edge or interior while not observed in other habitats included various representatives of the genera *Lasioglossum* and *Bombus*. The former are a particularly diverse genus, with 9 species unique to the forest interior and 5 to the forest edge, while the latter are larger-bodied and adaptable to cooler environments (forest sites sampled being higher altitude). *Bombus breviceps* in particular was abundant and confined to the forest interior. 4 of 13 *Ceratina* species were unique to the forests, while two *Coelioxys* and three *Nomia* species were unique to rubber plantations. Representatives of the genera *Xylocopa*, *Anthophora*, *Habropoda* were found only in the river bank, and diversity of *Lipotriches* were highest in those habitats.

The loss of suitable habitat (typically forest) is expected to drive extinction, increasing distance to neighbors (in a phylogenetic context), reducing phylogenetic diversity and increasing likelihood of sampling conspecifics. In the current analysis, the lowest phylogenetic relatedness scores NRI (an overall measure) and NTI (calculated from tree terminals only) were observed in the forest interior, and the highest in the old rubber plantation. Also, the forest interior (significantly) contrasted with the forest edge, with positive (clustering) for the interior and negative (overdispersion) at the edge. There is some debate as to whether mechanisms can be inferred based on these patterns. Clustering is thought to occur due to abiotic filtering^28^. Several mechanisms have been proposed to drive overdispersion, particularly, ‘facilitating interactions’, which are considered unlikely in the light-limited tropical forests^29^, and abiotic filtering in the case of radiation to different habitats^30^. Besides, we report a key that phylogenetic diversity and related indices are likely underestimated when calculated only on data from communities under study, which has implications for the field where comprehensive reference data are not normally included. This calls for approaches for rapid generation of comprehensive reference phylogenies in community phylogenetics, and builds on previous findings that inclusion of data from all study plots gives more accurate calculation of diversity indices^31^.

Comparisons of our findings with that of previous surveys of bees in NRWNNR^11^ were only partially consistent. It should be noted that the locations of habitat samples (within the NRWNNR) differed; ‘open’ sites used by ourselves might be more appropriately classified as river bank. Further, the forest sites surveyed herein were relatively high altitude compared to low altitude used by Meng *et al*. Insect communities often show variation in richness and composition along the elevational gradients of a mountain in response to differing environmental conditions^32^, although the elevational range observed in NRWNNR is modest. Still, evidence is strengthened for (at minimum) a unique bee fauna in the forest, with ‘indicator species’ found only in the forest by Meng *et al*., and greatest number of unique species herein, and a reduced diversity in aging rubber plantation in both studies. Most striking in contrast between the studies is greater number of species units reported in the current study. For example at least 25 *Lasioglossum* species in contrast to two reported by Meng *et al*., and a total count of 128 species in collections. This might be due to lack of distinctive morphological characteristics of the species and were only detected by the methods used in this study. On the other hand, we acknowledge the possibility of overestimated diversity herein, with error in DNA delineation tending towards oversplitting rather than lumping^33, 34^. Additionally with multi-gene delineation used herein, greatly less used in the literature (compared to delineation using single mitochondrial genes), the characteristics and error of which therefore being lesser known. Still, a significant advantage of study using standard DNA markers with widely used trapping technique is its amenability to further work, the data upon which inference is made being easily stored, accessed, re-analyzed and incorporated (into larger works) at any subsequent stage.

We make publicly available one of very few DNA datasets of Chinese bee communities, and present one of similarly few^11, 35^ studies on bee communities in tropical Xishuangbanna, China. Typical of tropical insects, the bees of NRWNNR are relatively diverse while being poorly described. Thus DNA sequences were invaluable in assisting delineation of species boundaries and derivation of community indices. The current work contributes to furthering knowledge of diversity of the bees of Xishuangbanna, while the integrative approach maximizes utility, ensuring data are incorporable into both taxonomic and DNA barcoding frameworks. We find certain impacts on bee phylogenetic diversity upon conversion to rubber plantation, which represent a threat to the fauna which are unique to natural areas. However, there are some characteristics of young plantations that make them amenable to bee activity. Further work is required to identify these, as their adoption could mitigate losses in biodiversity. Besides, the data presented in the current study helps lay the groundwork for understanding habitat-induced changes in diversity of pollinating insect in the understudied Asian tropics.

## Methods

All experimental protocols were approved by and carried out in accordance with the relevant guidelines and regulations of the Institute of Zoology, Beijing, China, and the University of Hohenheim, Stuttgart, Germany.

### Study Area

Fieldwork was conducted in the Naban River Watershed National Nature Reserve (NRWNNR, Fig. 1) in Xishuangbanna, a prefecture of southern Yunnan province, south China (22°10′ N and 100°38′ E). Climatic conditions allow for mostly tropical rain forest, representing the northernmost boundary of the humid tropics of Asia. However, other types of evergreen and seasonal forests exist depending on elevation and slope exposure. In recent decades, landscape transformation, and in particular the dramatic expansion of rubber monoculture cultivation, which now covers nearly a quarter of the land area of Xishuangbanna^36^, has greatly reduced natural forest cover, with an increase of forest fragments and a decrease of forest patch size. Remaining land cover types include secondary and primary forest fragments, grass and shrubland successions, banana plantations, and rice fields lower in the valley parallel to the river.

### Field Work

Insect collections covered both the dry and rain seasons of 2014 across keys habitats (young and old rubber plantation, natural forest interior and edge, open areas). Environmental recordings (temperature, humidity, windspeed etc.) were taken at collection time using a Kestrel handheld device. Insects were collected with Malaise traps into 100% ethanol. Disadvantages of this trap are limited spatial sampling, being relatively labor intensive to establish and fixed in nature, and bee capture rate can be lower than common alternatives such as pan trap and sweep net. Advantages are, as a passive flight intercept trap, investigator and capture bias is very low, and being the trap of choice for large-scale deployment in insect monitoring^37^, which in principle enables meta-analysis. 181 Malaise trap samples were collected, from 7th April until 19th August, during 2014 (collection details in Table 1 and Supplementary Table S1). Insects were collected (roughly) weekly, with at least two traps for each habitat (Fig. 1). There were some differences in sampling effort across habitat, traps were set a month later in the forest interior (missing a period of peak bee activity for that site), although four traps were set in the forest as opposed to two in other habitats. These differences were accounted for during data analysis (specifically, relevant analyses were repeated where omitting the initial month and using only two forest samples F1a and F2b). After transportation to the lab, preliminary sorting was conducted to morphospecies (Fig. 2a), a protocol requiring less time or expertise than taxonomic identification, and correlating well with taxonomic diversity^38^, and reducing redundancy during sequencing. From *each* of the 165 Malaise replicates (Table 1), 1–2 individuals from each morphospecies were selected with legs used for DNA extraction (Fig. 2b), hence since most species were caught across different weeks or in different locations, most species were sequenced several times. Bees were dried and mounted and taxonomic work conducted by author ZQ Niu, in reference to museum specimens. Vouchers (unique identifiers are given in Supplementary Dataset File) are held at the museum of the Institute of Zoology, Beijing, P. R. China.

Sample locations, placed in six key habitats (a) young rubber plantation, (b) old rubber plantation, (c) forest edge, (d) river bank and (e) natural forest, were further described by remote sensing based quantitative parameters regarding (a) elevation, (b) incoming solar radiation and (c) vegetation density. Elevation was extracted from a digital elevation model (Shuttle Radar Topographic Mission – SRTM) with a spatial resolution of 1 Arc-Second, (approx. 30 m). Incoming solar radiation was modeled based on the SRTM elevation model using the Solar Radiation toolbox in ArcGIS 10.3 software (ESRI). For the time between April 1^st^ and August 31^st^, 2014, the following parameters were modeled: (a) global radiation, i.e., amount of incoming (diffuse and direct) solar insolation in Watt hours per m² [Wh/m²], (b) incoming direct radiation [Wh/m²], (c) incoming diffuse radiation [Wh/m²] and (d) the duration of direct incoming solar radiation in hours [Wh/m²]. Vegetation density was characterized by the Normalized Difference Moisture Index (NDMI), an index which is frequently used in studies on vegetation dynamics^39, 40^ that is sensitive to vegetation leaf structure and water content^41^. NDMI was calculated based on a Landsat 8 OLI surface reflectance image with a spatial resolution of 30 m, acquired within the field sampling period on April 14^th^, 2014. NDMI values were averaged across a circular area of 120 m radius around each sample location to characterize vegetation density.

### Molecular work

Two loci were selected for characterizing bee diversity. The COI barcode fragment (585 bases) and 28S-rRNA (average 450 bases), the latter easily and reliably sequenced in the Hymenoptera^42, 43^, and having been applied for insect species delineation^44, 45^. Genomic DNA was extracted using QIAGEN DNeasy tissue extraction kit. The COI gene was amplified via PCR using LA Taq and 28 S using MightyAmp (both Takara). COI utilized the primer pairs LCO1490 (5′-GGTCA ACAAA TCATAA AGATA TTGG-3′) and HCO2198 (5′-TAAAC TTCAG GGTGA CCAAA AAATC A-3′)^46^ or COX-M1F (5′-TATCA ACCAA TCATAA AAATA TTG-3′) and COX-M2R (5′-TAAAC TTCTG GATGA CCAAA AAATC A-3′)^47^. The primer pairs D2-3549F (5′-AGTCG TGTTG CTTGA TAGTG CAG-3′) and D2-4068R (5′-TTGGT CCGTG TTTCA AGACG GG-3′)^48^ were used to amplify 28 S. All amplification reactions were performed in a total volume of 50 μl, with the COI reaction including 5 μl 10 × LA buffer, 5 μl MgCl2 (2.5 mM), 5 μldNTP (2.5 mM), 1 μl of each primer (10 mM), 0.5 μl LA Taq polymerase (5U/μl), 2–4 μl template DNA and distilled water to 50 μl. The 28 S reaction included 25 μl MightyAmp Buffer Ver.2,1 μl MightyAmp DNA Polymerase (1.25U/μl), 1 μl each primer (10 mM), 2–4 μl template DNA and distilled water to 50 μl. The PCR conditions were as following: 94 °C for 2 minutes, 35 cycles of 94 °C for 30 seconds, 48–50 °C for 50 seconds, 72 °C for 1 minute, and a final extension at 72 °C for 10 minutes for the COI reactions; 98 °C for 2 minutes, 35 cycles of 98 °C for 10 seconds, 58 °C for 15 seconds, 68 °C for 1 minute, and a final extension at 68 °C for 5 minutes for the 28 S reaction. Sequencing was performed with an ABI3130 sequencer. Sequences were aligned with Prank^49^ and checked visually using Bioedit^50^.

### Constructing a Reference Framework from Mined Data

A reference framework was constructed for various uses. Briefly, references were mined by downloading all DNA sequences for the Apoidea from NCBI, then sequence data for the two genes under current use were used as queries in a homology search using Blast + v2.2.28^51^. The Blast outputs were parsed with a Perl script and then filtered to retain a single sequence for each species, where we preferentially retained the member with the least ambiguous bases. Data filtration was carefully optimized to avoid omission of some locally (in China) sampled COI data that used non-standard primers (particularly Schaefer & Renner^52^).

### Delineating and Identifying OTUs

Species boundaries were arrived using an iterative and integrative approach. First, species-level delineation and taxonomy was obtained through iteration of morphological taxonomy and DNA clustering (Fig. 2c), which had the effect of more efficient taxonomic identification (which is the rate-limiting step). Initial morphological identifications were used to optimize species-level clustering of DNA sequences^53, 54^. For maximizing congruence, we used the Hubert and Arabie adjusted Rand Index (HA Rand Index), calculated in R^55^ using the Clues package^56^. To further assist in identification of errors during the iterative stage, broad-level (genus) taxonomic assignments were inferred with two-gene DNA barcoding, using the BAGpipe and SAP pipelines^57, 58^ querying the reference data mined earlier. Taxonomic iterations examined unexpected groupings, and various sources of error were corrected (sequence error, sequence label error, algorithmic shortcomings, specimen misidentification, labeling or data input error).

After a set of OTU and taxonomic names was established, they were treated as initial hypotheses to be tested using DNA taxonomy (Fig. 2d). Morphological groupings were overridden only where each DNA taxonomy method agreed otherwise. DNA taxonomy was conducted using both phylogenetic and phenetic means. Single threshold approaches were used in both cases, being both widely applied and showing no clear reduction in accuracy over more complex, multi-threshold models^59^. For phylogeny-based delineation, a RaxML tree^60^ was input into Poisson Tree Processes (PTP^61^). PTP is an implementation similar in principle to the widely-used General Mixed Yule Coalescent model (GMYC^62^). Both infer the locations of switches from intra-species to inter-species branching events, while the former method imposes fewer restrictions on input trees. A second intrinsic approach conducted was phenetic clustering of the molecular data to check for the presence of a ‘disjunct’ which is characteristic of the switch to species-level groupings^63, 64^. Distance-based clustering is typically conducted on single-gene data. Applying the approach to a concatenated dataset of two loci with distinct substitution characteristics required calculation genetic distances under distinct among-site variation models. The GTR + I + G model was preferred for both loci according to MrModelTest^65^. Distances were calculated using Paup*4b^66^. The genetic distances were used in hierarchical clustering (single linkage) in Esprit^67^, then we plotted genetic distance against the number of OTUs. The above phylogenetic and phenetic methods used two-genes. We additionally delineated based on COI only, since species-level characteristics of this marker have been robustly quantified. The barcode standard K2P distances were calculated for COI only, using PAUP*2b. We regard K2P distance of > 9% as exceptionally unlikely for members of the same species from a single region^15^.

### Impact of Land-Use on Bee Community Diversity in NRWNNR

Community structure at and between sample location was analyzed. Records for OTU presence over sites were input into R for calculation of diversity indices in the vegan R package^68^. Diversity was calculated at each site (alpha diversity), and compared among sites (beta diversity). Dissimilarity between pairs was quantified with the commonly used Bray-Curtis Distance, and average linkage hierarchical clustering conducted to describe relationships. Adonis and Anosim functions were used to test for significance in community composition between habitats, with environmental variables as alternative explanatory variables. The relation of environmental variables to composition of bee communities was determined through multivariate ordination, and multivariate structure was visualized with non-metric multidimensional scaling on the Bray-Curtis similarities. Additionally, we calculated beta diversity using the BAT package^21^, which takes incomplete sampling into account through rarefaction. Phylogeny-based metrics of community diversity were calculated in Phylocom v4.2^69^, using the OTU presence table and a RaxML tree of OTUs. Metrics were compared for the same set of members (the bee communities currently under study) but calculated in two different ways: using a phylogeny of only of these members, or from a phylogeny of these members and their relatives. For the latter, a comprehensive phylogenetic framework was constructed using the COI and 28 S reference data mined earlier, combined with new data, and a new phylogeny inferred with RaxML. For rapid generation of a reasonable tree-of-life using only two genes (limited information content), topological constraints were placed on refs 70, 71. The bee tree-of-life by Hedtke *et al*.^72^ was used for constraining the treesearch herein. The resulting tree was input into Phylocom for calculating diversity metrics of OTUs in NRWNNR bee communities.

The diversity observed in NRWNNR sites was compared with that of Meng *et al*.^11^, whose bee count data are publicly available (supplement 3.2 of Meng 2011^73^). Meng *et al*.’s data was re-analyzed, producing (non-phylogenetic) diversity scores as used by us. While the sampling locations within NRWNNR selected by ourselves and Meng *et al*. differed slightly, habitat types sampled in the two studies were regarded equivalent for the most part.

## Electronic supplementary material

supplement

Dataset 1
