# Supplementary material for: Integrative Profiling of Bee Communities from Habitats of Tropical Southern Yunnan (China)
Source: Sci Rep. 2017 Jul 13;7:5336. doi: 10.1038/s41598-017-05262-8 (PMC5509686; doi:10.1038/s41598-017-05262-8)

Supplementary Online Document for

Liu XW, Chesters D, Dai QY, Niu ZQ, Beckschäfer P, Martin K, Zhu CD.

‘Integrative Profiling of Bee Communities from Habitats of Tropical Southern Yunnan (China)’.

Contents

Supplementary Tables S1 a-m

Supplementary Figures S1 & S2

Also see Supplementary Dataset File

OTU_results_tables.xls: Excel format table of 3 sheets, showing OTU results organized by specimens (sheet 1), genera (sheet 2) and OTU (sheet 3)

Supplementary Table S1a: Details of Malaise collections. ManDian Young Rubber, YR1（location name in Chinese:曼点橡胶幼林）, GPS:22°07.890N, 100°40.447E, high:722M, accuracy: 9M. Note, information for Malaise samples is given irrespective of whether bees were caught.

date	Malaise sample ID	Windspeed /mps		Temperature /℃	wind chill	Humidity	Heat Stress Index	Dewpoint	Time	Note
		avg	max							
2014/4/1	Start									
2014/4/7	140407MA1001									
2014/4/14	140414MA1002	0.8	1.4	31.2	30.5	59	31.7	20.9	10:55	
2014/4/21	140421MA1003	0.9	1.6	31.5	32	61.3	38.4	23.4	11:00	
2014/4/27	140427MA1004	1.7	3.7	34.8	34.7	48	39.8-40.7	21.8	11:50	
2014/5/5	140505MA1005	1.7	3.6	31.6	31.6	67	33.3	23	11:05	
2014/5/14	140514MA1006	1.2	2.5	37	37	33.3	46.2	19.8	14:19	
2014/5/22	140522MA1007	3.0	9.2	36	35.9	55.2	32.0-52.2	24.1	15:02	
2014/5/29	140529MA1008	3.3	6.1	35	35	56.5-54.8	45.2-47.6	24.8	14:44	
2014/6/8	140608MA1009	1.4	2.0	34.6	34.6	66.8	40.1-44.8	29.8	12:28	
2014/6/17	140617MA1010	1.9	4.9	32.2	32.2	63.5	40.1-41.8	24.6	15:05	
2014/6/23	140623MA1011	4.3	7.7	38.3-38.9	38.8	48.4	NA	26.7-29.0	16:31	
2014/7/2	140702MA1012	0	0.8	30.3	30.3	100	42.8	30.2	12:31	
2014/7/9	140709MA1013	1.0	1.7	31.8	31.8	65.6-75.6	40.4	25.2	14:07	
2014/7/16	140716MA1014	2.0	3.1	30.6-34.9	31-34.9	72.4-74	40.0	26	13:02	
2014/7/25	140725MA1015	1.1	1.7	38.3-39.1	38.3	51.3-53.1	NA	27.1	15:05	
2014/8/19	140819MA1016	0.8	1.9	24.3	24.3	99.1	27.3	24.3	9:56	

Supplementary Table S1b: Man Dian Old Rubber, OR1（曼点橡胶成林）, GPS:22°07.957N, 100°40.379E, high:695M, accuracy: 15M Wass

date	Malaise sample ID	Windspeed /mps		Temperature /°C	wind chill	Humidity	Heat Stress Index	Dewpoint	Time	Note
		avg	max							
2014/4/1	Start									
2014/4/7	140407MB1001									
2014/4/14	140414MB1002	0.7	1.1	30	30.4	58.2	33.9	21.1	12:00	
2014/4/21	140421MB1003	1.4	2.5	34	33.7	57.5	38.5	23.2	12:25	
2014/4/27	140427MB1004	1.4	2.4	34	34	48.9	38.5	21.1	12:20	
2014/5/5	140505MB1005	1.2	1.8	29.2	29.2	64.5	33	22	11:35	
2014/5/14	140514MB1006	2.5	4.6	38.9	39.1	35.4	45.6	20.5	14:46	
2014/5/22	140522MB1007	2.5	4.1	30.3	30.6	64.3-66.8	36.2	23.5	15:35	
2014/5/29	140529MB1008	1.3	2.6	32.2	32.1	62.7	38.1	24.4	15:15	
2014/6/8	140608MB1009	0.9	1.6	32.3	32.3	69	40.8-42	26.2	12:46	
2014/6/17	140617MB1010	1.1	1.9	35.2	35.2	51.3-56.5	47.3	24.7	13:35	
2014/6/23	140623MB1011	1.8	4.0	35-36	35-36.2	48.8-52.1	40.9-46.2	23.8-24	14:56	
2014/7/2	140702MB1012	0	0	28.6-29.4	28.7-29.3	79-86.9	35.4-38.6	24.7-25.3	12:50	
2014/7/9	140709MB1013	0.6	1.4	29.4-33.3	29.4-33.3	66.6-75.9	35	24.6	14:25	
2014/7/16	140716MB1014	1.2	2.0	29.8	29.8	77.3	36.8	25	12:39	
2014/7/25	140725MB1015	1.3	2.7	30.9	30.9	71.3	39.1	25.2	14:50	
2014/8/19	140819MB1016	0.9	1.4	24.4	24.1	96.2	26.8	23.5	10:12	

Supplementary Table S1c: Man Fei Young Rubber, YR2（曼费橡胶幼林）, GPS:22° 10.016N, 100°40.126E, High: 760M, Accuracy: 22M Wass

date	Malaise sample ID	Windspeed / mps		Temperature /°C	wind chill	Humidity	Heat Stress Index	Dewpoint	Time	Note
		Avg	max							
2014/4/3	Start									
2014/4/10	140410MA2001									
2014/4/17	140417MA2002	0	0	23.4	23.4	65.3-69	24.1	17	9:55	
2014/4/23	140423MA2003	0.6	1.2	36.3	36.3	32.8-35	41.3	18.8	13:38	
2014/4/30	140430MA2004	0.8	1.2	28	28.1	65.3	30.8	20.8	11:15	
2014/5/8	140508MA2005	0	0	23.7	23.7	77.8	24.8	19.6	8:42	
2014/5/15	140515MA2006	1.4	2.8	34.7	34.7	48.3	40.6	21.6	12:50	
2014/5/22	140522MA2007	1.0	2.8	32.3	32.4	63.9	42.9	24.8	11:36	
2014/5/29	140529MA2008	1.2	2.3	27.3	27.3	79.1	31.7	23.7	9:00	
2014/6/8	140608MA2009	1.1	1.6	26	26	86.7	31.9	24	10:17	Rain
2014/6/17	140617MA2010	0.8	1.6	25.7	25.7	86.9	29.1	23.3	9:00	
2014/6/23	140623MA2011	0.9	1.2	29.9-30.9	29.9-30.1	70.2	35.9	23.9	10:42	
2014/7/2	140702MA2012	0	:0	27.3	27.3	84	31.6	24.2	10:14	
2014/7/9	140709MA2013	0.7	1.1	27.4	27.4	86.5	33.1	24.9	9:32	
2014/7/16	140716MA2014	0.9	2.4	26.8	26.9	91.7	29.7	23.9	10:31	
2014/7/25	140725MA2015	0.9	1.2	25.1	25.1	90.3	28	23.2	9:50	
2014/8/19	140819MA2016	0	0	26.4	26.4	94.3	29.4	24.6	12:20	

Supplementary Table S1d: Man Fei Old Rubber, OR2（曼费橡胶成林）, GPS:22° 10.104N, 100°40.777E, High: 671M, Accuracy: 45M Wass

date	Malaise sample ID	Windspeed / mps		Temperature /°C	wind chill	Humidity	Heat Stress Index	Dewpoint	Time	Note
		avg	max							
2014/4/3	Start									
2014/4/10	140410MB2001									
2014/4/17	140417MB2002	0.7	1.2	31.9	32.1	53.3	36.8	21.2	13:40	
2014/4/23	140423MB2003	2.2	4.1	37.3	37.3	34	40.2	18.4	14:17	
2014/4/30	140430MB2004	0.8	1.7	25.3	26.6	69.1	29.2	20.1	10:41	
2014/5/8	140508MB2005	0	0	23.6	23.7	86.5	24.9	21	9:20	
2014/5/15	140515MB2006	1.9	3.6	32.5	32.5	55.4	37	22.4	12:10	
2014/5/22	140522MB2007	2.3	4.1	31	30.8	64.4	35.4	23.4	10:25	
2014/5/29	140529MB2008	1.8	2.6	32.3	32.4	57.1	38.3	22.6	11:15	
2014/6/8	140608MB2009	0	0	26.9	27	83.6-86.9	31.7-32.4	23.9-24.7	9:42	rain
2014/6/17	140617MB2010	0.9	2.2	30.7	30.7	68	3.9-37.3	24.4	11:55	
2014/6/23	140623MB2011	1.9	2.7	31.6-32.6	31.9-32.7	66.1-62.4	39.2-39.8	24.1	12:19	
2014/7/2	140702MB2012	0	0	24.9	24.9	92.2	28.5	23.6	9:20	
2014/7/9	140709MB2013	0	0	26.9	26.9	85.3	31.9	24.3	9:05	
2014/7/16	140716MB2014	0	0	26.6	26.8	86.6	28.9	23.1	9:42	
2014/7/25	140725MB2015	0	0	26.1	26.1	83.1	29.3	22.8	9:05	
2014/8/19	140819MB2016	0	0	26	26.1	94	31.1	24.9	11:09	

Supplementary Table S1e: Guo Men Shan Forest Edge, FE1（过门山林缘）, GPS: N22.24804° E100.59247°, High: 671M, Accuracy: 45M Wass

date	Malaise sample ID	Windspeed / mps		Temp /℃	wind chill	Humidity	Heat Stress Index	Dewpoint	Time	Note
		avg	max							
2014/4/2	start									
2014/4/8	140408MC1001									
2014/4/15	140408MC1002	0.9	1.6	28.2	29.6	62.1	34.1	21.3	12:25	
2014/4/22	140421MC1003	0.0	0.9	25.7	25.6	67	26.3	18.8	10:35	
2014/4/28	140428MC1004	0.0	0.0	27	26.9	66.5	30.4	20.5	10:55	
2014/5/6	140506MC1005	0.8	0.8	26.5	26.5	75	28.1	21.3	12:20	
2014/5/13	140506MC1006	0.8	3.4	32.2	32.2	40.8-46.8, 42.9	32.9	17	14:45	
2014/5/20	140520MC1007	0.6	1.2	32.8	32.8	55.2	39.3	22.8	12:58	
2014/5/27	140527MC1008	1.0	1.1	30.1	30.1	69.5	36.6	24.3	14:30	
2014/6/6	140606MC1009	0.9	1.1	29.6	29.6	69.1	34.7	23	10:37	
2014/6/12	140408MC1010	1.3	2.1	28.9	28.9	69.4	33.1	23	14:10	
2014/6/19	140619MC1011	0.0	0.9	27.5	27.6	78	32.1-32.3	23.6	13:10	rain
2014/6/25	140625MC1012	0.0	0.0	25.3	25.3	81.4	28.4	22.1	10:10	
2014/7/4	140703MC1013	0.0	0.8	27.2	27	78.8-84.6	31.7	23.8	11:30	rain
2014/7/10	140710MC1014	0.0	0.0	31	31	77.6	39.6	26.6	15:45	
2014/7/17	140717MC1015	0.8	1.4	28.4	28.4	77.8-80.7	33.7	24.2	12:50	
2014/7/26	140726MC1016	0.0	0.0	29.5	29.5	83.3-86.2	34.2	25.3	13:37	
2014/8/18	140818MC1017	0.0	0.0	29.4-29.6	27.8-29.5	78.4-92.4	31.2-36.8	25.3-25.6	13:34	

Supplementary Table S1f: Ban Qian Di Forest Edge, FE2（搬迁地林缘）, GPS:22 °14.049N, 100 °36.931E, High：943M, Accuracy: 15M

date	Malaise sample ID	Windspeed / mps		Temperature /°C	wind chill	Humidity	Heat Stress Index	Dewpoint	Time	Note
		avg	max							
2014/4/2	Start									
2014/4/8	140408MC2001									
2014/4/15	140415MC2002	0.9	2.5	27.4	27.5	58.4	29.2	18.5	10:30	
2014/4/22	140422MC2003	0.7	1.2	38.7	38.5	44	49.8-50.6	23.9	13:10	
2014/4/28	140428MC2004	0.8	1.3	28.2	28.2	63.1	31	20.6	12:05	
2014/5/6	140506MC2005	0.0	0.0	24.9	24.9	84.8	28.4	21.6	13:30	
2014/5/13	140506MC2006	2.4,	3.8	31.5	31.5	56.4	36.7	21.6	11:00	
2014/5/20	140520MC2007	1.0	1.8	33.5	33.5	56.2-64.2	48.6	25.1	10:30	
2014/5/27	140527MC2008	1.0	1.4	34.2	34.2	69.7-71.2	52.9-62	30.7	10:25	
2014/6/6	140606MC2009	1.0	1.9	31.8	31.8	73.3-75.8	42.7	26.6	14:34	
2014/6/12	140612MC2010	0.0	0.0	28.7	28.7	81.3	34.9-30.6	23.6-26	9:53	
2014/6/19	140619MC2011	0.0	1.0	27.4	27.4	84.3	31.7-32.9	24.8-25.2	10:12	rain
2014/6/26	140626MC2012	0.0	0.8	28.2	28.2	77.3	33	23.5	13:45	
2014/7/3	140703MC2013	1.3	2.0	28.8-29.3	28.9-29.3	74.4	34.5	24.1	14:05	
2014/7/10	140710MC2014	1.3	1.7	26.7	26.7	78.8-79.2	30.8	23	10:00	
2014/7/17	140717MC2015	1.1	1.5	29	29.1	80.6	40.4	26.4	11:00	
2014/7/26	140726MC2016	0.8	1.8	37.5	37.5	61.2	54-57.1	28.7	14:42	
2014/8/18	140818MC2017	0.6	1.3	30.5	30.5	80.5	40.2	26.6	14:37	

Supplementary Table S1g: An Ma Xin Zhai River Bank, RB1（安麻新寨河边）, GPS:22 °11.824N, 100 °38.788E, High: 708M, Accuracy: 11M

date	Malaise sample ID	Windspeed / mps		Temperature /℃	wind chill	Humidity	Heat Stress Index	Dewpoint	Time	Note
		avg	max							
2014/4/3	start									
2014/4/9	140409MD1001									
2014/4/16	140416MD1002	1.2	2.4	28.4	28.3	60.5	32.9-33.3	20	10:17	
2014/4/24	140424MD1003	1.7	3.8	35.5-40.2	40.3	43.9-49.1	49.8-54.3	25	13:15	
2014/4/29	140429MD1004	0.7	1.2	23.2	23.2	79.2-83.3	24.7	19.4	8:50	
2014/5/7	140507MD1005	1.1	1.9	25.1	25.1	81.1	27.4	21.9	12:37	
2014/5/14	140514MD1006	1.3	2.8	31.5	31.5	52.9	36.3	20.9	10:33	
2014/5/21	140521MD1007	3.3	5.7	31.8	31.8	68.7	39	24.7	11:00	
2014/5/28	140528MD1008	2.8	5.9	35.6-39.6	35.6-39.6	51.6-58.9	NA	28.7-29.7	11:50	
2014/6/4	140604MD1009	3.1	5.2	34.9	35	60.6	44.2-48.1	24.5-25.3	11:30	
2014/6/11	140611MD1010	0.7	1.5	31.9	31.7	71.9	38.1-44	26.2	11:30	
2014/6/18	140618MD1011	2.2	3.8	25.5	25.5	88.1-90.2	29-32	24.1	10:35	
2014/6/27	140627MD1012	4.2	9.4	33.5	32.2	61.4	47.7	25.6	12:47	
2014/7/3	NA	1.1	2.2	27.1	27.1	83.2	33.2	24.4	10:45	
2014/7/11	140711MD1013	0.8	1.6	30.3	30.3	74.1	37.3	25.1	9:55	
2014/7/18	140718MD1014	1.5	2.4	24.9	24.9	94.2	28.2	23.9	9:33	
2014/7/25	140725MD1015	0.8	1.5	26.8	26.8	85.5	32.5	24.2	10:50	
2014/8/19	140819MD1016	2.6	4.1	25	25	100	30	27.8	15:41	

Supplementary Table S1h: Da Nuo You River Bank, RB2（大糯有河边）, GPS:22 °12.772N, 100 °38.105E, High: 826M, Accuracy: 25M

date	Malaise sample ID	Windspeed / mps		Temperature /°C	wind chill	Humidity	Heat Stress Index	Dewpoint	Time	Note
		avg	max							
2014/4/9	start									
2014/4/16	140416MD2001	1.3	2.9	32.8-33.8	34	44.5	39.2	21.3	13:36	
2014/4/24	140424MD2002	0.7	1.3	33	32.9	52.5	37.4-38.5	21.8-22.3	10:50	
2014/4/29	140429MD2003	0.0	0.0	24.2	24.2	68.2-76.8	24.5	19.2	9:20	
2014/5/7	140507MD2004	0.0	0.0	25	25	80-91.2	25.6	21.5	11:55	
2014/5/15	140515MD2005	1.0	1.4	25.4	25.4	73.6	28.5	20.6	9:30	
2014/5/21	140521MD2006	3.0	6.1	31	31.1	62.1	35.6	23	13:10	
2014/5/28	140528MD2007	1.6	5.3	35.5	35.5	49.1	48.2	24.9	15:10	
2014/6/4	140604MD2008	1.1	2.1	39.4-40.1	39.7	46.4	52.4	25-26.9	12:50	
2014/6/11	140611MD2009	1.4	3.0	37.8	38.2	53.5	NA	27.3	13:03	
2014/6/18	140618MD2010	0.9	2.4	25.3	25.7	87.7	28.5-29.2	23.3	11:45	
2014/6/27	140627MD2011	1.2	3.9	31.9-32.6	31.8-35.6	57.8-68.3	44.6-49.2	26.7	14:33	
2014/7/3	140703MD2012	1.4	2.1	26.4	26.4	82.7	32.3	23.9	12:40	
2014/7/4	NA	1.6	2.7	29.4	29.4-29.8	72	36.1	24.3	12:16	
2014/7/11	140711MD2013	0.0	0.0	27.0-27.6	27	86-89	31-32.6	25.2	10:11	
2014/7/18	140718MD2014	1.0	1.6	27.8	27.9	81.1	34.1	24.4	11:30	
2014/7/25	140725MD2015	0.9	1.9	29.3	29.3	81.2	37.5-38.0	26.3	16:26	
2014/8/18	140818MD2016	1.6	2.7	29.4	29.4-29.8	72	36.1	24.3	12:16	

Supplementary Table S1i: Xiao Nuo You Shang Zhai Natural Forest, F1-A（小糯有上寨天然林）, GPS: 22 °13.469N, 100 °35.469E, High: 1661M, Accuracy: 53M

date	Malaise sample ID	Windspeed / mps		Temperature /°C	wind chill	Humidity	Heat Stress Index	Dewpoint	Time	Note
		avg	max							
2014/5/8										
2014/5/12	NA	0.0	0.0	24.6	24.6	77.8-78.4	25.8	19.7	11:45	
2014/5/19	140519ME1-A001	1.1	2.5	30.3	27.7	63.5	31.5	21.3	13:10	
2014/5/26	1405140523IE2-A00226E1-A002	0.0	1.0	27.9	27.9	76.9-79.6	25.8-26.7	20.7-21.1	12:27	
2014/6/3	140603ME1-A003	1.0	2.8	33.5	33.5-35.1	51.8-55.0	36.3-42.5	22.4-23	12:20	
2014/6/9	140609ME1-A004	0.0	0.7	22.2	22.3	96.6	23	21-21.3	12:31	Rain
2014/6/16	140616ME1-A005	0.0	0.0	24.5	24.5	79.6	26.6	21	10:55	Rain
2014/6/24	140624ME1-A006	0.6	1.1	24.4-25.5	25.6-27.3	71.2	30	21.4	10:30	
2014/7/1	140701ME1-A007	0.0	0.9	24.4-26.9	24.4	80.4	29	22-22.4	12:55	
2014/7/8	140708ME1-A008	0.0	0.0	23.3	23.3	87.6	25.1	21.4	11:30	Heavy rain
2014/7/15	140715ME1-A009	0.0	0.0	25.6-26.1	25.6	75.5	24.2-26	20.2	12:55	Heavy rain
2014/7/21	140721ME1-A010	0.0	0.0	21.5-22.3	20.7-21.9	86.1-89.6	21.5	19.1	12:38	Heavy rain
2014/7/26	140726ME1-A011	0.0	0.0	26.7	26.7	79.2	26.7	21.8	10:16	
2014/8/18	140818ME1-A012	0.0	0.0	22.0-22.9	21.8	99.8	23.7	21.8	10:17	

Supplementary Table S1j: Xiao Nuo You Shang Zhai Natural Forest, F1-B（小糯有上寨天然林）, GPS: N22.22638 °, E100.59414 °, high: 1602 m, West 1 m

date	Malaise sample ID	Windspeed / mps		Temperature /℃	wind chill	Humidity	Heat Stress Index	Dewpoint	Time	Note
		avg	max							
2014/5/12										
2014/5/19	140519ME1-B001	0.0	0.8	28.3	28.3	69.3	32.4	22.1	14:46	
2014/5/26	140526ME1-B002	0.0	0.0	26.7	26.7	75.6-81.1	27.1	21.1	13:45	
2014/6/3	140603ME1-B003	0.0	0.0	34.5	34.2	54-57.6	35.2-38.3	21.9-23.3	14:00	
2014/6/9	140609ME1-B004	0.0	0.0	24.2	24.2	85.1	26.5	21.6	13:14	
2014/6/16	140616ME1-B005	0.0	0.0	22.3	22.4	88.3	23.7	20.4	12:05	
2014/6/24	140624ME1-B006	0.0	0.0	23.7	23.8	84.2	25.9	21	11:52	
2014/7/1	140701ME1-B007	0.0	0.0	25.1-26.1	25.1-26.1	81.9	26.8-28.2	21.9	14:25	
2014/7/7	140708ME1-B008	0.0	0.0	24.3	22.8-24.3	80.5-90.5	23.6	21.2	12:20	slight rain
2014/7/15	140715ME1-B009	0.0	0.0	22.6	22.6	89.7	24.3	21	13:55	heavy rain
2014/7/21	140721ME1-B010	0.0	0.0	20.5	20.4	100	21.8	20.8	13:05	heavy rain
2014/7/26	140726ME1-B011	0.0	0.0	26.6	26.6	77.6	26.6	22	10:50	
2014/8/18	140818ME1-B012	0.0	0.0	25.1	25.1	87.1	27.6	22.6	10:59	

Supplementary Table S1k: Beng Gang Natural Forest, F2-A（蚌岗天然林）, GPS: 22 °06.682N, 100 °35.056E, high:1872M, accuracy: 20M Wass

date	Malaise sample ID	Windspeed / mps		Temperature /℃	wind chill	Humidity	Heat Stress Index	Dewpoint	Time	Note
		avg	max							
2014/5/9	start	2.3	4.5	20.3	20.3	75.7	20.8	15.9	13:55	slight rain
2014/5/16	140516ME2-A001	1.7	6.3	23.8	23.8	65	24.5	16.4	10:55	
2014/5/23	140523ME2-A002	0	0	26.1	26.1	70.2	24.7	19.3	10:45	
2014/5/30	140530ME2-A003	2.4	3.5	22.4	22.4	78.2	23.0	18.3	10:00	
2014/6/7	140607ME2-A004	1.9	3.0	21.6	21.6	84.8	23.2	19.1	9:52	
2014/6/13	140613ME2-A005	1.0	1.6	18.8	18.9	89.8	19.4	17.3	10:00	heavy rain
2014/6/20	140620ME2-A006	2.2	3.3	19.2-21.4	19.2-21.5	81.8-89.6	19.4-21.5	17.8-18.0	9:50	slight rain
2014/6/30	140630ME2-A007	0	0	20	20	87.1-91.8	20.2	18.8	10:31	
2014/7/7	140707ME2-A008	1.1	2.2	21	20.9	89.6	22.5	19.3	10:17	
2014/7/14	140714ME2-A009	1	1.9	22.7	22.8	83.8	24.3	19.6	10:39	rain
2014/7/24	140724ME2-A010	0.9	1.4	21.8	20.6-21.8	84.5	21.8-22.4	18.9	10:55	
2014/8/17	140817ME2-A011	1.7	2.4	19.4	19.5	93.3	20	18.6	11:26	rain

Supplementary Table S1l: Beng Gang Natural Forest, F2-B（蚌岗天然林）, GPS: 22°06.657N, 100°35.083E, High: 1866M, accuracy:14Wass

date	Malaise sample ID	Windspeed / mps		Temperature /°C	wind chill	Humidity	Heat Stress Index	Dewpoint	Time	Note
		avg	max							
2014/5/16	start	1.0	2.1	28.4	28.4	56.6	30.4	18.9	13:48	
2014/5/23	140523ME2-B001	1.0	2.7	22.4	22.6	75.8	22.4-25.3	18.2	11:30	
2014/5/30	140530ME2-B002	0.9	2.3	26.4	26.6-27	66.5	28.7	19.8	10:48	
2014/6/7	140607ME2-B003	0.8	1.5	20.2	20.3	88	20.5	18.1	10:18	
2014/6/13	140613ME2004	0.7	2.2	17.6	17.6	91.1-95.3	18.2	16.8	10:30	heavy rain
2014/6/20	140620ME2-B005	1.5	3.5	18.4	18.4	94.2-96.5	18.8	17.6	10:16	rain
2014/6/30	140630ME2-B006	0	0	22.1	22.1	83.9	22.8	19.2	11:01	rain
2014/7/7	140707ME2-B007	1.7	2.8	20.2	20.2	94.5	21.8	19.7	10:55	
2014/7/14	140714ME2-B008	0.9	1.2	19.7	19.7	96.4	19.3	18.1	11:15	rain
2014/7/24	140724ME2-B009	1.1	2.3	20.6	19.5	97.1	20.1	19.1	11:35	
2014/8/17	140817ME2-B010	1.6	2.1	17.4	17.4	100	18.1	17.4	12:00	rain

Supplementary Table S1m: Na Ban Station, CK（纳板站）, GPS: N22.16725, E100.65869, high: 721M

date	Malaise sample ID	Windspeed / mps		Temperature /℃	wind chill	Humidity	Heat Stress Index	Dewpoint	Time	Note
		avg	max							
2014/4/2	start									
2014/4/8	140408MCK001									
2014/4/18	NA									
2014/4/30	140430MCK002									
2014/5/22	140522MCK003	0.9	2.7	35.1	35.1	56.7	44.6	25.3	14:25	
2014/6/18	140618MCK004	0.8	1.6	29.9-30.3	30.2	76.7-81.7	36.2-39.1	24.9-26.3	16:25	
2014/7/10	140710MCK005	2.7	5.0	32.7	32.7	61.5	49.8-42	24.7	17:00	
2014/7/25	140725MCK006	2.2	2.7	33.3-33.9	33.4	70.2	48.4	27.9	14:05	
2014/8/19	140819MCK007	1.2	1.9	24.7	24.8	100	29	26.3	13:10	

Supplementary Figure S1: Phenetic clustering analysis. Genetic distances calculated using under a GTR model, gamma distributed rates, and partitioned for two-loci. Upper: number of OTU generated under varying genetic distance. Lower: taxonomic Rand Index (tRI) describes amount of congruence between clustered sequences and the taxonomic names they were independently assigned. Congruence is calculated under varying clustering parameters (0.000 to 0.020). Optimal clustering parameter, that in which maximal congruence is observed, is depicted by vertical bar.

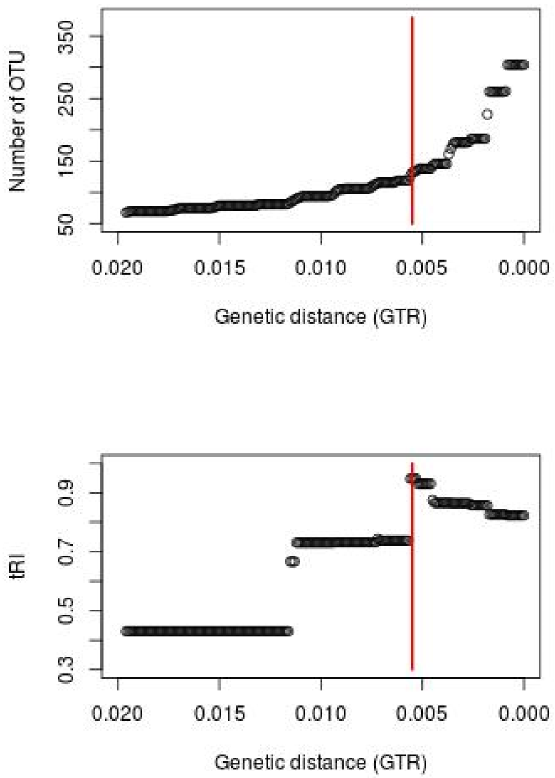

Supplementary Figure S2: PTP results. Within species branches are colored red, and between species branches are blue. Notable error is found in species *Apis cerana*, the inclusion of a single long branch (to two specimens) results in overestimation of species diversity. Whole tree is made available in Newick format as supplementary dataset file.

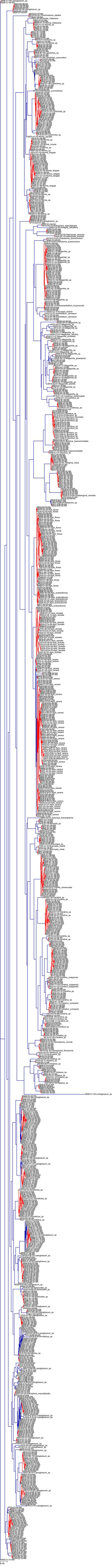

Supplement: Supplementary file 1 — supplement [file 41598_2017_5262_MOESM1_ESM.pdf]
